# Four screening instruments for frailty in older patients with and without cancer: a diagnostic study

**DOI:** 10.1186/1471-2318-14-26

**Published:** 2014-02-26

**Authors:** Ineke HGJ Smets, Gertrudis IJM Kempen, Maryska LG Janssen-Heijnen, Laura Deckx, Frank JVM Buntinx, Marjan van den Akker

**Affiliations:** 1 Department of Clinical Epidemiology, VieCuri Medical Centre, Venlo, The Netherlands; 2 Department of Health Services Research, CAPHRI School for Public Health and Primary Care, Maastricht University, Maastricht, The Netherlands; 3 Department of Epidemiology, GROW School for Oncology and Developmental Biology, Maastricht University Medical Center, Maastricht, The Netherlands; 4 Department of General Practice, KU Leuven, Leuven, Belgium; 5 Department of General Practice, CAPHRI School for Public Health and Primary Care, Maastricht University, Maastricht, The Netherlands

**Keywords:** Frailty, Screening, Geriatric assessment, Cancer

## Abstract

**Background:**

Frailty in older patients might influence treatment decisions. Frailty can be determined using a Comprehensive Geriatric Assessment (CGA), but this is time-consuming and expensive. Therefore we assessed the diagnostic value of four shorter screening instruments.

**Methods:**

We tested the abbreviated CGA (aCGA), the Vulnerable Elders Survey-13 (VES-13), the Groningen Frailty Indicator (GFI) and the Geriatric 8 (G8). A full CGA including functional status, cognitive status, depression, nutrition and comorbidity was used as reference. A minimum of 85% for both sensitivity and specificity was predefined as acceptable. Data were collected through personal interviews by trained interviewers. We assessed people aged ≥ 70 years: 108 patients with recently diagnosed cancer recruited in hospitals and 290 without cancer recruited by general practitioners in the Netherlands and Belgium.

Frailty was defined as having impairment in at least two domains of the full CGA. We used original cut-offs for the screening instruments and calculated sensitivity, specificity, positive and negative diagnostic values and the percentage classified as frail.

**Results:**

Sensitivity of aCGA was 79% and 87% for patients with and without cancer; specificity was 59% and 64%. Sensitivity of VES-13 was 67% and 82% for patients with and without cancer; specificity was 70% and 79%. Sensitivity for GFI was 76% (in both groups) and specificity 73% (in both groups). Sensitivity for G8 was 87% and 75% for patients with and without cancer; specificity was 68% (in both groups).

**Conclusions:**

No screening instrument was acceptable according to our predefined minimum of 85% for both sensitivity and specificity. The diagnostic value of the investigated instruments is rather poor and one could wonder about their additional value to clinical judgment.

## Background

An increase in the number of older people is leading to an increase in the number of patients diagnosed with cancer [1]. Older people are a very heterogeneous group with respect to comorbidity and social and psychosocial functioning [2]. This heterogeneity may complicate treatment decisions [3,4]. Therefore, treatment of older patients demands a specific approach and the presence of frailty should be taken into account.

Frailty is an ambiguous concept. Fried and colleagues associated frailty with dependency, institutionalization and mortality [5,6], but it is also associated with physiological age [2]. From another perspective, frailty is considered to be a multidimensional concept that is influenced by biological and physiological factors but also by personal characteristics and environment [7]. In addition, frailty is generally considered to be a potentially reversible state, which makes its diagnosis important [8,9].

Frailty can be determined by a Comprehensive Geriatric Assessment (CGA) [10]. The International Society for Geriatric Oncology (SIOG) recommends performing a CGA for all patients diagnosed with cancer aged ≥ 70 years [11]. However, a CGA is time-consuming and therefore expensive. An alternative could be the use of validated shorter screening instruments that indicate whether further assessment of geriatric problems using a full CGA might be relevant [12].

In the present study, four screening instruments for frailty were compared with a full CGA: the abbreviated CGA (aCGA) [13], the Vulnerable Elders Survey-13 (VES-13) [14], the Groningen Frailty Indicator (GFI) [15] and the Geriatric 8 (G8) [16,17]. These instruments were selected because they are often used in daily care to assess frailty. Although the validity of the selected screening instruments has been studied previously, evidence about psychometric quality is still limited. A recent study by Hamaker and colleagues [18] compared eight screening instruments to a full CGA and found limited discriminative power with a wide range in sensitivity (25–92%) and specificity (39–100%). Other recent studies concluded that screening tools had too little predictive power [19,20] to identify frailty. In some studies [21], only older patients with cancer were included to investigate shorter screening instruments. In other studies [22], only older people without cancer were included. Identification of frailty is important for both groups, however, the diagnostic value of screening instruments might be different in older cancer patients as many frailty-related symptoms such as fatigue and nutrition are influenced by (treatments of) cancer.

We aimed to identify screening instruments that best assess the risk of frailty in people aged ≥ 70 years with and without cancer. Since there is still no generally accepted standard of frailty [18], we decided that having at least two out of five positive scores on the domains that are part of the full CGA (i.e., functional status, cognition, depression, nutritional status and medication use) was an indication of frailty.

## Methods

This study was part of the KLIMOP study [23], which is a large prospective cohort study conducted at the universities of Leuven and Hasselt in Belgium and Maastricht in The Netherlands. It is performed in collaboration with the Limburg Cancer collaboration foundation (LIKAS), 7 hospitals, 13 general practices in the Netherlands and 31 in Belgium. The target population of the KLIMOP study consists of older cancer patients aged ≥ 70 years, older patients aged ≥ 70 years without a previous diagnosis of cancer and cancer patients between 50–69 years. For each group a sample size of 360 participants per country was proposed, enabling within-country analyses.

The aim of the KLIMOP study is to assess the impact of cancer, aging and their interaction on the well-being of older cancer patients [23].

### Participants

For this study two groups were defined. One group consisted of people aged ≥ 70 years who were recently given a primary diagnosis of breast, prostate, lung or gastrointestinal cancer. This group was recruited at the oncology wards of five Belgian and two Dutch hospitals. The other group consisted of people aged ≥ 70 years without cancer. This group was recruited through general practices in Belgium and The Netherlands. Both groups were recruited between June 2010 and December 2012. Exclusion criteria were the inability to speak Dutch, a formal diagnosis of dementia, a previous diagnosis of invasive cancer (except non-melanoma of the skin), being too ill to participate or a life expectancy shorter than six months (based on the judgment of the attending doctor) [23]. We collected data from 509 participants.

### Data collection

All participants were assessed by trained interviewers. Participants were screened for frailty by means of the aCGA, VES-13, GFI, G8 and a full CGA. The questions from the different screening instruments were asked in a personal interview and the same questions were not asked twice. The full CGA consists of five domains: functional status, cognition, depression, nutritional status and medication use. Functional status was measured by Activities of Daily Living (ADL) using the Barthel Index [24] and by Instrumental ADL using the Lawton IADL-scale [25]. Cognitive status was assessed by the Mini Mental State Examination (MMSE) [26]. Depressive symptoms were measured using the Geriatric Depression Scale (GDS-15) [27]. Nutrition was assessed by food intake (QOL) [28] and weight loss (GFI) [15]. Medication use, as reported by the participants, was included as an indication for morbidity.

### Instruments and cut-off values

The cut-off values of the four selected screening instruments were derived from previous studies (Table 1). The aCGA [13] consists of 15 questions covering three domains: functional status (seven questions on ADL and IADL), cognitive status (four questions from the MMSE) and depression (four questions from the GDS-15). A cut-off value was identified for each domain, indicating whether a more elaborate assessment was needed for that domain: ≥ 1 for ADL and IADL; ≤ 6 for the MMSE; and ≥ 2 for the GDS-4 [21]. A need for further assessment of frailty was indicated if one of the aCGA domains scored positive.

**Table 1:** Cut-off values for the screening Instruments aCGA, VES-13, GFI, G8, and for the full CGA

**DOMAINS**	**aCGA**		**VES- 13**		**GFI**		**G8**		**Full CGA**	
	**Cut-off maximum**		**Cut-off maximum**		**Cut-off maximum**		**Cut-off maximum**		**Cut-off maximum**	
**Age**				3				2		
**Functional status**	≥ 1	7		7		5		4	≥ 2	19
**Cognitive status**	≤ 6	8				1		2*	≤ 23	30
**Depression**	≥ 2	4				5		2*	≥ 8	15
**Nutrition**						1		8		
- Food intake									< 2	2
- Weight loss									< 3	3
**Comorbidity**										
- Medication use						3		1	>3	
**INDICATION FOR FRAILTY**	**Positive score on ≥1 domain**	**None**	**≥ 3**	**10**	**≥ 4**	**15**	**≤ 14**	**17**	**Positive score on ≥ 2 domains**	**None**

*Cut-off maximum of 2 for cognitive status *or* depression.

The VES-13 includes questions about age, self-rated health-status, physical fitness and need for assistance with activities. It consists of 13 questions with a maximum score of 10 points. We used the original cut-off value of ≥ 3 as an indication of frailty [14].

The GFI assesses mobility, physical fitness, assistance needed with toileting and shopping, poor hearing and vision, medicine use, complaints about memory and depression. It consists of 15 questions with a maximum score of 15 points. The original cut-off value of ≥ 4 was used to indicate frailty [15].

The G8 consists of eight questions about age, functional status, cognitive status, nutrition and medication use. The maximum score is 17 points. The original cut-off value of ≤ 14 was used for indicating frailty [17]. The specific G8 questions were not part of the interview; instead, the answers were extracted from similar questions asked at different parts of the interview.

The full CGA conducted in this study consists of five domains: functional status, cognition, depression, nutritional status and medication use. We used separate cut-offs for each domain: a problem on at least two items of the functional domain (ADL and IADL), a score of ≤ 23 on the MMSE or a score of ≥ 8 on the GDS-15. The ‘nutrition’ domain was considered positive if food intake declined during the last week [28] or if there had been a loss of at least one kilogram in weight over the last three months [17]. For ‘medication use,’ we defined a cut-off score of > 3 drugs. We used a proxy outcome for the full CGA and considered frailty to be present if two or more of the five domains scored positive.

### Statistical analysis

For statistical analysis we used Statistical Package for the Social Sciences (SPSS) version 19. We first described the demographic and cancer-related clinical characteristics of the participants (Table 2). To assess the diagnostic value of the different screening instruments, the sensitivity (correctly classifying frail participants as positive), specificity (correctly classifying non-frail participants as negative), the negative predictive value (NPV, proportion of non-frail participants according to the full CGA in those with a negative screening test result) and the positive predictive value (PPV, proportion of frail participants in those with a positive screening test result) of the scores were calculated together with their 95% confidence intervals (CI). When there was no overlap in scores among the instruments (taking the CI into account), differences were considered to be statistically significant. To estimate the clinical usefulness of each screening instrument, we calculated the percentage of patients who each instrument classified as frail (and who thus had to be referred for a full CGA) in addition to the PPV.

**Table 2:** Demographic and clinical characteristics of the patients

**Variable**	**Patients aged ≥ 70 years with cancer**		**Patients aged ≥ 70 years without cancer**	
	**N**	**%**	**N**	**%**
**All**	108		290	
- Male	38	35	105	36
**Median age (range)**		76 (70–88)		78 (70–97)
**Living situation**				
- Not alone (partner or children)	73	67	197	68
- Alone	31	29	88	30
- Nursing home	0	0	0	0
- Others	4	4	5	2
**Cancer diagnosis**				
- Lung	7	6	0	0
- Breast	51	47	0	0
- Gastrointestinal	45	42	0	0
- Prostate	5	5	0	0

Furthermore, we varied the cut-off values of the four screening instruments to investigate whether they resulted in better outcomes. We first started with the cut-off values as proposed in the original studies; thereafter, the cut-off value was increased and decreased by one point.

Finally, diagnostic values were calculated separately for patients diagnosed with breast cancer and patients diagnosed with gastrointestinal cancer, using the original cut-off values.

For screening purposes a high sensitivity is generally considered as the most important diagnostic characteristic of an instrument. It is important for correctly diagnosing frailty so that an individual treatment plan can be developed. However, a high specificity is also important to avoid an unnecessary expensive and burdensome full CGA. Therefore, we a priori defined a score of 85% or higher for both sensitivity and specificity as adequate.

### Ethical considerations

As part of the KLIMOP study, this study was approved by the Ethical Review Board of the Catholic University (KU) Leuven and the University Hospitals Leuven (S52097 - ML6279) (Belgium) and by the Medical Ethics Committee of the Maastricht University Medical Centre (NL31414.068.10) (The Netherlands). We applied good clinical practice guidelines procedures (GCP) such as the principles of the Declaration of Helsinki (October 2008 version), the Belgian law of 7 May 2004 concerning clinical trials with humans and Dutch laws including the Medical Research Involving Human Subjects Act and the Personal Data Protection Act. Participants were only included after giving written informed consent.

## Results

Data were originally collected from 509 patients, but 111 were excluded because of incomplete data. Participants excluded from the analysis were not different from those included in the analysis regarding sex. They were slightly older (mean 78,9 and 77,2, respectively, p = 0.033) and more often had a diagnosis of cancer (54% and 18%, respectively, p < 0.001).

Among these excluded patients, we were missing values for the full CGA from 61 patients, for the aCGA from 58 patients, for the G8 from 19 patients, for the VES-13 from 12 patients and for the GFI from 59 patients.

Finally, data from 398 patients were available for analyses. The population characteristics are shown in Table 2. The included population consisted of 108 patients with cancer and 290 without cancer. About 65% were female and about 35% were male. The male–female ratio was similar in patients with and without cancer. In both groups most of the patients did not live alone (68%). In the group with cancer, breast cancer (43%) and gastrointestinal malignancy (49%) were most represented. The results of the four screening instruments are shown in Table 3.

**Table 3:** **Diagnostic values in % (95**% **CI between brackets) for frailty for patients aged ≥ 70 years with and without cancer**

	**With and without cancer (%)**	**With cancer (%)**	**Without cancer (%)**	**With and without cancer (%)**	**With and without cancer (%)**
**aCGA**	**CUT-OFF ≥ 1**			**CUT-OFF ≥ 2**	
Sensitivity	85 (81 – 88)	79 (71 – 87)	87 (83 – 91)	58 (53 – 63)	
Specificity	63 (58 – 68)	59 (50 – 68)	64 (59 – 70)	94 (91 – 96)	
PPV	68 (64 – 73)	64 (55 – 73)	70 (65 – 75)	90 (87 – 93)	
NPV	82 (78 – 85)	75 (67 – 83)	84 (80 – 88)	70 (66 – 75)	
Classified as frail	60	59	61	31	
**VES-13**	**CUT-OFF ≥ 3**			**CUT-OFF ≥ 2**	**CUT-OFF ≥ 4**
Sensitivity	78 (74 – 82)	67 (58 – 76)	82 (77 – 86)	81 (77 – 85)	74 (70 – 78)
Specificity	76 (72 – 80)	70 (61 – 78)	79 (74 – 83)	69 (65 – 74)	78 (74 – 82)
PPV	75 (71 – 80)	67 (58 – 76)	78 (73 – 83)	71 (67 – 76)	76 (72 – 80)
NPV	78 (74 – 82)	70 (61 – 78)	82 (77 – 86)	79 (75 – 83)	76 (72 – 80)
Classified as frail	50	48	51	55	47
**GFI**	**CUT-OFF ≥ 4**			**CUT-OFF ≥ 3**	**CUT-OFF ≥ 5**
Sensitivity	76 (71 – 86)	79 (61 – 87)	74 (69 – 79)	88 (84 – 91)	59 (54 – 63)
Specificity	73 (68 – 77)	71 (63 – 80)	73 (68 – 78)	49 (44 – 54)	83 (79 – 87)
PPV	72 (68 – 77)	72 (63 – 80)	72 (67 – 78)	62 (57 – 67)	76 (72 – 81)
NPV	76 (72–80)	78 (71 – 86)	75 (70 – 80)	81 (77 – 85)	68 (63 – 73)
Classified as frail	51	53	50	69	37
**G8**	**CUT-OFF ≤ 14**			**CUT-OFF ≤ 13**	**CUT-OFF ≤ 15**
Sensitivity	78 (74 – 82)	87 (80 – 93)	75 (70 – 80)	61 (56 – 65)	96 (94 – 98)
Specificity	68 (63 – 72)	64 (55 – 73)	69 (64 – 74)	86 (82 – 89)	39 (34 – 44)
PPV	70 (65 – 74)	69 (61 – 78)	70 (64 – 75)	80 (76 – 84)	60 (55 – 65)
NPV	77 (73 – 81)	84 (77 – 91)	75 (70 – 80)	70 (65 – 74)	91 (88 – 94)
Classified as frail	55	60	52	37	78

### aCGA

Sensitivity was 85%, specificity was 63%, PPV was 68% and NPV was 82%. The overall accuracy was 74%: 164 patients (41%) were correctly classified as frail and 129 patients (33%) were correctly classified as not frail. The aCGA classified 60% of the patients as frail, compared to 48% with the full CGA. Sensitivity and NPV were lower (79% and 75%, respectively) in the group with cancer compared to the group without cancer (87% and 84%, respectively). There were no significant differences in specificity, PPV or classification as frail between the two groups. Using a higher cut-off value (≥ 2) for the aCGA led to fewer classifications of frailty (31%) and less symmetric scores on sensitivity (58%) and specificity (94%).

### VES-13

Sensitivity was 78%, specificity was 76%, PPV was 75% and NPV was 78%. The overall accuracy was 77%: 150 patients (38%) were correctly classified as frail and 156 patients (39%) were correctly classified as not frail. The VES-13 classified 50% of the patients as frail, compared to 48% with the full CGA. In the group with cancer, all diagnostic values were significantly lower compared to the group without cancer. The percentage of patients classified as frail was lower in patients with cancer (48%) compared to those without cancer (51%). Using a lower cut-off value (≥ 2) for the VES-13 led to more classifications of frailty (55%) and less symmetric scores on sensitivity (81%) and specificity (69%). Using a higher cut-off value (≥ 4) led to fewer classifications of frailty (47%) and somewhat less symmetric scores on sensitivity (74%) and specificity (78%).

### GFI

Sensitivity was 76%, specificity was 73%, PPV was 72% and NPV was 76%. The overall accuracy was 74%: 146 patients (37%) were correctly classified as frail and 149 patients (37%) were correctly classified as not frail. The GFI classified 51% of the patients as frail, compared to 48% with the full CGA. There were no differences in diagnostic values between patients with and without cancer. The percentage of patients with cancer who were classified as frail (53%) was higher than in the group without cancer (50%). Using a lower cut-off value (≥ 3) for the GFI led to more classifications of frailty and less symmetric scores on sensitivity (88%) and specificity (59%). Using a higher cut-off value (≥ 5) led to fewer classifications of frailty and less symmetric scores on sensitivity (59%) and specificity (83%).

### G8

Sensitivity was 78%, specificity was 68%, PPV was 70% and NPV was 77%. The overall accuracy was 73%: 151 patients (38%) were correctly classified as frail and 139 patients (35%) were correctly classified as not frail. The G8 classified 55% of the patients as frail, compared to 48% with the full CGA. Sensitivity and NPV were significantly higher (87% and 84% respectively) in the group with cancer compared to the group without cancer (75% and 75% respectively). There were no significant differences in specificity and PPV between the two groups. The percentage of patients classified as frail in the group with cancer (60%) was higher than in the group without cancer (52%). Using a lower cut-off value (≤ 13) led to fewer classifications of frailty (37%) and less symmetric scores on sensitivity (61%) and specificity (86%). Using a higher cut-off value (≤ 15) led to more classifications of frailty (78%) and less symmetric scores on sensitivity (96%) and specificity (39%).

### Stratified analysis

Sensitivity and specificity of the different screening tools had largely overlapping confidence intervals for patients with breast cancer, patients with gastro-intestinal cancer, and the total group of cancer patients. Only for patients with breast cancer the sensitivity of the VES-13 was higher (75% vs. 67%), as was the specificity of the G8 (74% vs. 64%). For patients with gastro-intestinal cancer, specificity of the G8 was lower (43% vs. 64%).

## Discussion

We evaluated the diagnostic value of four screening instruments (aCGA, VES-13, GFI and G8) using the CGA as the reference standard to identify frailty in people aged ≥ 70 years with and without cancer. No screening instrument was acceptable according to our predefined minimum of 85% for both sensitivity and specificity. There were differences in diagnostic values between the groups with and without cancer, except for the GFI. Changing the cut-off points did not lead to more appropriate results. This is disappointing because there is a big need for good quality screening instruments in everyday care for older people.

### Comparison with previous research

Previous research outcomes are available but the results vary widely [18]. Different results occurred in part because of differences in the definition of the full CGA in previous studies, relating both to content and to cut-off scores for domains. There were also differences in the included study populations like age, presence of cancer and type of cancer. Our study looked at older people with and without cancer, which did not result in large differences between these groups. The cancer patients who were included in our study were recently diagnosed with a primary breast, prostate, lung or gastrointestinal cancer. The selected instruments used in this study, excluding the GFI [19], had previously mostly been evaluated among older patients with different types of cancer and were not validated in elderly patients without cancer.

Our study found sensitivity scores for the aCGA (85%) and the GFI (76%) that were higher than those reported in the study by Hamaker and colleagues [18]. This higher sensitivity can be explained by using five instead of three domains of the CGA as a reference standard or by the fact that we also included people without cancer. Next in our study, elderly patients with cancer scored lower (79%) on sensitivity for the aCGA but higher (87%) on sensitivity for the G8 compared to patients without cancer. This difference might be caused by the predominance of nutrition in the G8 and the fact that malnutrition might be more prevalent in cancer patients [29]. In addition, we only included patients in the cancer group who were recently diagnosed and not yet treated, and therefore who were still in relatively good health. This might have influenced the results.

The higher (76%) sensitivity score that we found for the GFI as compared with the review of Hamaker and colleagues [18] cannot be explained by differences in the study population (with and without cancer) as our GFI results were almost similar in patients with and without cancer. The sensitivity and specificity values that we found for the VES-13 and G8 all fell within the wide range that Hamaker and colleagues [18] presented in their review. A recent study by Biganzoli and colleagues [30] investigated the cardiovascular health study instrument (CHS) and the VES-13 in elderly cancer patients. They concluded that the right screening tool is still missing because of the great variability in specificity observed between subgroups that differed in disease status (early or advanced) and type of early cancer. This limits its applicability to the general population. Still, no good quality screening instrument scoring ≥ 85% on both sensitivity and specificity has been found. A review by Ruiz and colleagues [31] also concluded that there is a need for a shorter reliable tool for rapid and complete assessment.

### Strengths and limitations

This study has several strengths. First, the study design enabled us to provide external validation and comparison of different screening instruments in the same population and using the same reference standard. Second, we compared elderly patients with and without cancer and were therefore able to study the robustness of outcomes in older persons from different samples. Third, we evaluated different cut-off values for the screening instruments.

A study limitation is that we used a reference standard based on the five most common CGA domains. However, no consensus exists about which instruments or other tests should be used for a full CGA [18]. Therefore, a “gold” standard does not exist. A second limitation refers to the operationalization of frailty using a full CGA. We defined frailty as impairment on at least two domains of the CGA. However, there is no consensus about the definition and operationalization of frailty yet. Using our definition, 48% of the participants were identified as frail. If we would have used impairment on at least one domain as cut-off for frailty, 73% of the participants would have been classified as frail.

A third limitation is the predefined minimum of 85% for both sensitivity and specificity. One could argue that for screening instruments a high sensitivity might be preferred as it is crucial to minimize ‘false negatives’. However, a high specificity can also be considered important because of costs for the organization and burden for the patient. No literature was found about final thresholds so we chose these cut-offs which may be arbitrary. A fourth limitation is the lack of information about morbidity and cancer stage. Unfortunately, this information is not available yet. A final limitation refers to the exclusion of patients with missing values. Excluded patients were slightly older and more likely to have cancer. As our results are presented for cancer and non-cancer patients separately, this cannot have biased our results.

### Implications of these study results

Sensitivity is important for selecting vulnerable patients for a full CGA and ruling out the possibility of frailty. Frailty is a state that might be fully or partly reversible with the help of an individual treatment plan [8] including interventions that can reverse or prevent it [9].

Diagnosing frailty and creating an individual treatment plan might prevent complications and preserve a patient’s autonomy. Hence, from the patient’s point of view, the aCGA can be recommended for elderly patients without cancer (sensitivity of 87%) and the G8 can be recommended for elderly patients with cancer (sensitivity of 87%). Specificity is important for correctly selecting fit individuals for whom no full CGA is indicated. This prevents a patient from having to undergo an unnecessary complete CGA, creating less of a burden for the patient and reducing healthcare costs. However, none of the screening instruments in this study showed a satisfying specificity.

Taking these advantages for the patient and for health care costs into account, a screening instrument with high diagnostic values of sensitivity and specificity is preferred. Therefore, we aspired to a score of at least 85% for both sensitivity and specificity in order to use the instrument as a screening tool for correctly selecting vulnerable people for a full CGA. Nevertheless, we did not identify any high scores on both sensitivity and specificity in any of the available screening instruments included in this study. The diagnostic value of the investigated instruments was rather poor and one could wonder about their additional value to clinical judgment.

### Further research

The ultimate goal of screening for frailty is to predict outcomes with respect to functioning and treatment. Much more research will be necessary to develop a screening instrument that has appropriate diagnostic values, that is suitable for distinguishing between frail and fit patients and that can be used as a supporting tool for treatment decisions. Second, since some differences in diagnostic values exist between patients with and without cancer and between patients with different types of cancer, further research is needed to investigate whether specific diseases need specific screening instruments to be more predictive. Additionally, there is an urgent need for longitudinal studies on the diagnostic value of instruments for mid- and long-term outcomes on factors like functional status, institutionalization or mortality.

## Conclusions

No screening instrument was acceptable according to our predefined minimum of 85% for both sensitivity and specificity. The diagnostic value of the investigated instruments is rather poor and one could wonder about their additional value to clinical judgment.

## Competing interests

The authors declare that they have no competing interests.

## Authors’ contributions

IS contributed to the study concept and design, data acquisition, analyses and interpretation, manuscript preparation, editing and review. GK, MJH, FB and MvA contributed to the study concept and design, data analyses and interpretation, manuscript editing and review. LD participated in the acquisition, analyses and interpretation of the data and contributed to the editing and review of the manuscript. All the authors have given final approval of the version to be published.

## Pre-publication history

The pre-publication history for this paper can be accessed here:

http://www.biomedcentral.com/1471-2318/14/26/prepub
